# Quincke’s disease: an unusual pathology

**DOI:** 10.1093/jscr/rjad085

**Published:** 2023-03-04

**Authors:** Gabriel Atan Sanchez, Madison Boot, Abdul Lathif

**Affiliations:** Wagga Wagga Base Hospital, Wagga Wagga, Ears Nose and Throat Department, NSW, Australia; Wagga Wagga Base Hospital, Wagga Wagga, Ears Nose and Throat Department, NSW, Australia; Wagga Wagga Base Hospital, Wagga Wagga, Ears Nose and Throat Department, NSW, Australia

## Abstract

A 50-year-old male presents to the emergency department in rural Australia with a sore throat, globous sensation of his oropharynx and a swollen uvula. Within the previous 12 months, this was his third and most severe presentation of Quincke’s disease. In all instances, it was aggravated by cold weather. His airway was not compromised. He was admitted under the Ears, Nose and Throat (ENT) specialist and managed with 200 mg of intravenous hydrocortisone, followed by regular intravenous dexamethasone as well as paracetamol for analgesia. He improved over 12 h and was discharged with 1 week of steroids. He followed up with the ENT specialist in the community. A cause could not be found. He was subsequently consented and booked for a partial uvulectomy.

## INTRODUCTION

The term *Quincke’s disease* is used to describe a phenomenon where a patient experiences isolated angioedema of the uvula [1]. It is a rare clinical diagnosis and as a consequence, its epidemiology has not been documented in the literature [5]. The causes of this disease are variable and include allergies, trauma (including intubation), inhalation exposure, general anaesthesia, infections and hereditary angioedema [1, 2]. Most commonly, the cause is that of a type I immediate hypersensitivity reaction, as seen in those with specific allergies [3]. However, in patients with recurrent episodes, like the one described in this report, a high degree of suspicion should be given to the presence of a complement system disorder such as C1 esterase inhibitor deficiency or hereditary angioedema [4].Although a rare condition, Quincke’s disease has the potential to be life threatening if the airway becomes compromised due to an isolated enlarged uvula. Therefore, it is an important condition to be aware of, and also the health professionals benefit from being prepared for the worst-case scenario in such patients.

## CASE REPORT

A 50-year-old Australian Indigenous male presented to the emergency department with a 1-day history of sore throat and a globous sensation associated with a swollen uvula. The patient awoke from sleep with acute onset dyspnoea due to worsening uvula swelling. He was unable to speak due to pain and had difficulties breathing when supine. He denied cough, fevers, nausea or vomiting. There were no obvious precipitants to this event.

The patient had three previous, less severe episodes of uvular swelling in the past 12 months. All previous episodes came on randomly, aggravated by the cold weather, and resolved spontaneously without antibiotics or steroids. He has a past medical history of gastroesophageal reflux disease and obstructive sleep apnoea. He takes omeprazole daily and is awaiting his continuous positive airway pressure machine. The patient has a history of allergies including urticaric rash to bee stings and tongue swelling to honey but does not require epinephrine for management. There was no family history of angioedema or uvula swelling. The patient is a lifelong non-smoker and is up to date with all immunizations.

**Figure 1:**
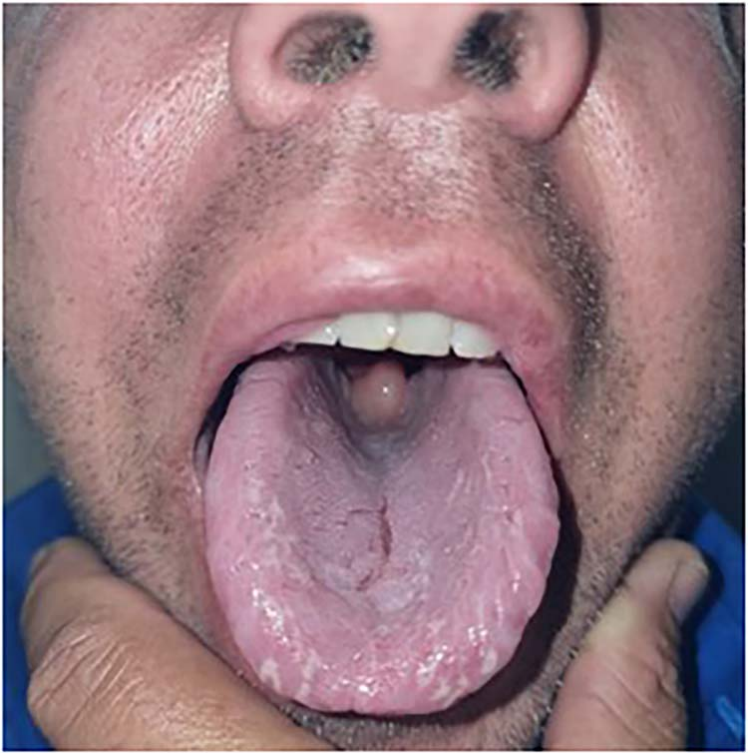
Oral examination revealing an enlarged pendulous uvula with pale mucosa and no midline deviation.

**Figure 2:**
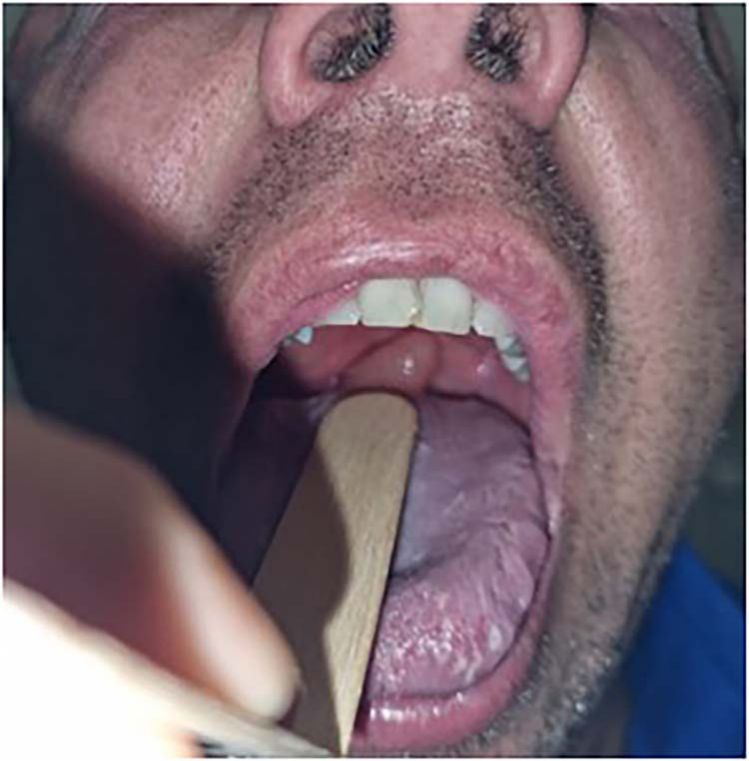
Closer inspection of uvula on oral examination.

On examination, the patient’s observations were within normal limits and he was afebrile. There was no obvious stertor or stridor. Oral examination revealed an enlarged pendulous uvula with pale mucosa and no midline deviation (Figs 1 and 2). There was no erythema or swelling of the palate-pharyngeal arches. The patient was able to protrude his tongue, with no trismus or floor of mouth tenderness. The neck examination was unremarkable with no submandibular tenderness or cervical lymphadenopathy. The patient’s white cell count was 8.1 × 10^9^/L and the C reactive protein was less than 3 mg/L. All other bloods were within normal limits.

The patient was diagnosed with Quincke’s disease and admitted under the Ears, Nose and Throat (ENT) specialist. He was managed with an immediate dose of 200 mg intravenous hydrocortisone, then regular intravenous dexamethasone 8 mg three times a day and oral paracetamol for pain relief. Over 12 h there was an immediate improvement of the uvula oedema, allowing the patient to speak full sentences and tolerate a full diet. The patient was discharged home later that day with a 1-week course of steroids, regular analgesia and follow-up with an ENT specialist.

On review by the specialist ENT surgeon, he was found to have an elongated and bulky uvula. His C1 Esterase Inhibitor Level was negative. After discussion with the patient he was consented and booked for a partial uvulectomy.

## DISCUSSION

The examination on our patient revealed an isolated finding of angioedema of the uvula; however, a specific cause could not be found. His history of previous episodes discloses an association with cold weather and given that he presented to us in winter, this is a possible explanation. In the literature relating to Quincke’s disease, however, we could not find any cases related to cold exposure. Furthermore, given his history of multiple episodes, he should be investigated for a complement system disorder. Unfortunately, blood tests to suggest this, such as C1 esterase and mast cell tryptase levels [5], were not taken during his emergency department admission.

In patients with a clinical suspicion of Quincke’s disease, investigations should be sought. Suggested work-up includes C1 esterase and mast cell tryptase levels, swab cultures of the pharynx and uvula, blood cultures, latex agglutination studies looking for *Haemophilus influenzae* type B (HIB) and *Streptococcus pneuomniae* antigens (in both blood and urine), lateral neck radiographs and toxicology of both blood and urine [6].

The primary concern when a patient presents with Quincke’s disease is a threatened airway. If the airway is compromised, then initial management must be focused on restoring a safe airway [7]. Medical treatment for Quincke’s disease includes the administration of the following (as monotherapy or in combination): adrenaline; diphenhydramine; cimetidine; corticosteroids [2]. If a corticosteroid is given, then dexamethasone seems to be the most appropriate choice due to its potent anti-inflammatory properties and long half-life [8]. Surgical management can also be considered. Symptoms tend to resolve within 24 to 48 h [5]. Given that our patient has had recurrent episodes, this being his third, follow-up with an ENT surgeon was recommended for further investigation of a cause.

A differential diagnosis to consider in patients with suspected Quincke’s disease is that of uvulitis. This entity is infectious in nature and can be associated with epiglottitis, pharyngitis or tonsillitis. It is treated with antibiotics, whereas Quincke’s disease is not [7, 8]. If uvulitis due to epiglottitis is suspected, then direct visualization or a lateral neck radiograph should be considered for further investigation [8]. Other differentials to consider in such patients include retropharyngeal and peritonsillar abscess [6].

## CONCLUSION/LEARNING POINTS

(i) Quincke’s disease can be a life-threatening condition if the airway becomes compromised due to an enlarged uvula [4].
(ii) Quincke’s disease should be differentiated from infective uvulitis. The latter is infectious in nature and is associated with epiglottitis, pharyngitis and tonsillitis. Infective uvulitis is treated with antibiotics, whereas Quincke’s disease is not [6, 7].
(iii) Causes include trauma, inhalation exposure, for example, marijuana, general anaesthesia, medication reaction (angiotensin converter enzyme inhibitors), infections and hereditary angioedema.
(iv) Immediate treatment of uvular edema depends on the degree of airway compromise. In emergencies, intravenous H1 and H2 blockers, corticosteroids and even epinephrine may be necessary [9]. In these cases, early involvement of anaesthetist or otolaryngology team is necessary to help facilitate endotracheal intubation, and cricothyroidotomy is necessary.
(v) Surgical management suggested includes uvular debulking with uvulotomy or uvulectomy
